# Uncovering the surge: dengue fever on the rise

**DOI:** 10.3389/fepid.2025.1478425

**Published:** 2025-02-25

**Authors:** Noah Wheaton, Christian Wong, Huda Gasmelseed, Samrawit Zinabu, Aseem Sood, Rithika Rajendran, Madison Shead, Amaya Sanders, Tabitha Norton, Miriam Michael

**Affiliations:** ^1^ Department of Internal Medicine, Howard University, Washington, DC, United States; ^2^ Department of Infectious Diseases, Howard University, Washington, DC, United States; ^3^ Department of Internal Medicine, University of Maryland School of Medicine, Baltimore, MD, United States; ^4^ Department of Oncology, Washington DC Veterans Affairs Medical Center, Washington, DC, United States

**Keywords:** dengue fever, global warming, vector transmission, Aedes mosquito, viral infection

## Abstract

**Introduction:**

Dengue fever, traditionally a tropical disease, has shown a notable increase in incidence within the United States over recent decades. This paper focuses on the increase in dengue fever cases in Maryland during increasing temperature and humidity and the expanding geographical range of Aedes mosquitoes, the primary vectors for dengue virus transmission.

**Methods:**

Electronic health data was used to identify patterns in dengue incidence from 2014 to 2024. Correlation analysis between temperature and dengue incidence and a review of humidity factors was conducted.

**Results:**

Results indicate an increased incidence of dengue fever cases over the past decade. However, a reduction in dengue incidence was observed in 2019–2020, likely due to COVID-19-related travel restrictions.

**Discussion:**

As global temperatures increase, the habitats suitable for Aedes mosquitoes have expanded, allowing for their proliferation in previously inhospitable regions. Additionally, higher temperatures can accelerate the life cycle and viral replication rates in these mosquitoes, further enhancing transmission potential. Humidity, another key environmental factor, influences the life expectancy of dengue mosquitoes. This research underscores the urgency of addressing climate change as a public health issue, emphasizing the need for integrated vector management strategies and public health preparedness to mitigate the growing threat of dengue in temperate regions. By understanding the interplay between global warming, humidity, and dengue transmission, we can better inform policy decisions and healthcare practices to curb the spread of this disease in Maryland and the United States.

## Introduction

Dengue fever is a mosquito-borne illness that traditionally occurs in tropical and subtropical regions. The dengue viruses, which are members of the Flaviviridae family, come in four different serotypes: DENV-1, DENV-2, DENV-3, and DENV-4 (1). This variety explains the range of illness severity, from asymptomatic infections to dengue fever (DF), dengue hemorrhagic fever (DHF), and dengue shock syndrome (DSS). The *Aedes aegypti* and *Aedes albopictus* mosquitoes transmit the virus from human to human, making it the fastest-spreading mosquito-borne disease worldwide (2, 3). However, most cases are localized to Asia, the Western Pacific regions, the Americas, and the Eastern Mediterranean, where it is endemic in over 100 countries (4). The first cases in the Americas were reported in the 1600s, and since then, global warming and increased international travel have led to a steady rise in cases. In 2023, the World Health Organization (WHO) Region of the Americas reported 4.5 million cases and 2,300 deaths (4).

While the incidence of dengue fever has been predominantly concentrated in tropical regions, cases have also been reported in the continental United States, including autochthonous (locally acquired) cases. A study examined the geographic distribution and dispersal of *Aedes albopictus* in the United States from its initial detection in the United States in Texas in 1987 to a decade later, revealing that the species had expanded to 678 counties across 25 states (5). Since 2001, at least three dengue outbreaks have been reported in the US, occurring in Florida, Texas, and Hawaii (6). In 2017, the CDC estimated that it was very likely that the potential range was both Aedes aegypti or A. Albopictus mosquitoes could live and reproduce, including Maryland (7). Two recent studies corroborate the CDC's prediction. Faiman et al. and Rothamn et al. observed the *Aedes aegypti* and *Aedes albopictus* mosquitoes, respectively, were present in Maryland. Providing additional evidence that these mosquitoes have established themselves and are capable of survival in Maryland (8).

Most cases involve asymptomatic recent travelers. However, symptoms can rapidly escalate, with DF being the most common. Clinical manifestations of DF include arthralgia, rash, headache, myalgia, fever, retro-orbital pain, and hemorrhagic signs (9). Abnormal liver function tests, low platelet counts, and gastrointestinal symptoms have also been reported in association with DF (9). To diagnose dengue fever acutely, the Centers for Disease Control and Prevention (CDC) recommends testing for dengue virus non-structural protein-1 (NS1) and IgM or using nucleic acid amplification tests (NAAT) alongside IgM tests (10). A positive NAAT test confirms the viral infection. The CDC advises that if dengue is suspected, providers should ensure adequate management while diagnostic testing is underway (10).

There is currently no cure for dengue infection; treatment consists of supportive care, including acetaminophen, fluid repletion, and blood transfusions if the patient is hemorrhagic (3). Viremia can last up to twelve days so patients with an acute infection should avoid mosquito bites (11). Despite DF's vast and variable symptoms, the consistent rise in cases within the District of Columbia, Maryland, and Virginia (DMV) of the United States calls for a proactive approach to deepen our understanding of the disease and the reasons behind this upward trend. This paper explores the increase in dengue fever cases within the DMV area and proposes plausible causes for this concerning trend.

## Materials and methods

This study is a retrospective cohort analysis investigating the incidence of dengue fever in Maryland using electronic health records (EHRs). De-identified data were extracted from the Epic EMR system over a 10-year period (July 26, 2014, to July 25, 2024). The primary objective was to assess trends in dengue incidence in relation to environmental factors, particularly temperature and humidity, across the state of Maryland.

### Data source

The University of Maryland's Epic SlicerDicer tool was used to collect de-identified clinical and epidemiological data. SlicerDicer is a data extraction tool that provides information on large patient populations, enabling efficient analysis of trends and diagnoses within the health system. Although 89% of acute care hospitals nationwide used Epics software we only used data of patients from the state of Maryland (12).

### Study population

The base population included 5,866,616 patients from the University of Maryland Health System during the study period. Patients diagnosed with dengue fever were identified using ICD-10 codes A90 (Dengue fever) and A91 (Dengue Hemorrhagic Fever), and further validation was conducted using positive IgG and IgM serological test results.

### Temperature and humidity data

Temperature and humidity data were sourced from statewide time series records. These records provided yearly trends in peak temperature (in Fahrenheit) and peak humidity levels (as a percentage) for the study period.

### Geographic area

Salisbury, Maryland, a major population center on Maryland's Eastern Shore with a humid subtropical climate, was selected as a key location for assessing environmental variables.

### Variables

Yearly peak temperature and peak humidity levels were analyzed to evaluate their relationship with dengue incidence.

### Time frame

The analysis covered a 10-year period from 2014 to 2024, aiming to detect the incidence correlating with dengue incidence.

### Data slicing

The initial cohort comprised all patients over the 10-year study period. The data were then sliced by the diagnosis of dengue fever using ICD-10 code A90 and A91. Afterward, the dataset was further segmented by the presence of positive IgG and IgM test results to confirm cases of dengue fever. The incidence of dengue was calculated relative to the population at risk in Maryland.

## Statistical analysis

Statistical analyses were performed using GraphPad Prism 10.0. Year, temperature, and humidity were included as primary covariates in relation to dengue fever incidence. Linear regression was applied to assess the strength of trends, while multivariate analyses were used to isolate the impact of these independent variables. Results are presented in tables and graphs to enhance clarity and interpretation.

1. Linear Regression Analysis
  ○ Linear regression models were applied to explore the associations of each covariate, temperature and humidity with dengue fever incidence. Analyses were conducted both including and excluding the pandemic years (2020 and 2021) to account for any anomalies during that period. Each model's R-squared values, F-statistics, and *p*-values were evaluated to assess the strength and statistical significance of these factors as potential predictors of dengue incidence.
2. Multivariate Logistic Regression Analysis
  ○ A multivariate logistic regression model was employed to identify independent predictors of dengue fever incidence, with temperature and year included as covariates. Analyses were conducted both including and excluding the pandemic years (2020 and 2021). This approach allowed for an assessment of each factor's unique contribution to dengue incidence

### Ethical considerations

This study was deemed exempt from full board review by the University of Maryland Institutional Review Board, as all data used were de-identified. Informed consent was waived due to the retrospective nature of the study and the use of anonymized data.

## Results

Our study analyzed 5,866,616 patients within the EMR system and identified 119 patients diagnosed with dengue fever between 2014 and 2024. Among these patients, 11 tested positive for IgG antibodies, while another 11 tested positive for IgM antibodies. Among 119 patients, 40.6% were female, and 59.4% were male. The age distribution showed that 6.3% of cases occurred in individuals below 18 years old, 65.6% in individuals aged 18–60, and 28.1% in those above 60 years old.

The Eastern Shore region of Maryland, characterized by a humid subtropical climate and bordered by the Chesapeake Bay and the Atlantic Ocean, was found to have a disproportionately high incidence of dengue cases. Although this area comprises only 7.4% of Maryland's population, 24% of dengue cases were from the Eastern Shore, suggesting environmental factors might contribute to elevated case rates in this region.

The prevalence of dengue fever increased over the study period, with annual cases as follows: 7 in 2014, 8 in 2015–2017, 10 in 2018, 13 in 2019, 9 in 2020, and 10 in 2021. Despite the COVID-19 lockdown, which was expected to result in fewer cases due to restricted travel, the reduction in incidence was not as pronounced as anticipated. Following the lockdown, cases increased, with 11 in 2022, 15 in 2023, and 20 in the first six months of 2024.

Annual Prevalence of dengue fever and total Maryland population for the year (13, 14, 15)

• 2014: 7 cases         5,960,064
• 2015: 8 cases         5,988,528
• 2016: 8 cases         6,007,014
• 2017: 8 cases         6,028,186
• 2018: 10 cases         6,042,153
• 2019: 13 cases         6,045,680
• 2020 (COVID pandemic): 9 cases 6,177,224
• 2021 (post-pandemic): 10 cases  6,177,224
• 2022: 11 cases         6,163,981
• 2023: 15 cases         6,180,253
• 2024 (first 6 months): 20 cases   6,180,253

### Incidence rates

Incidence Rates per 100,000 Population.

**Figure 1:**
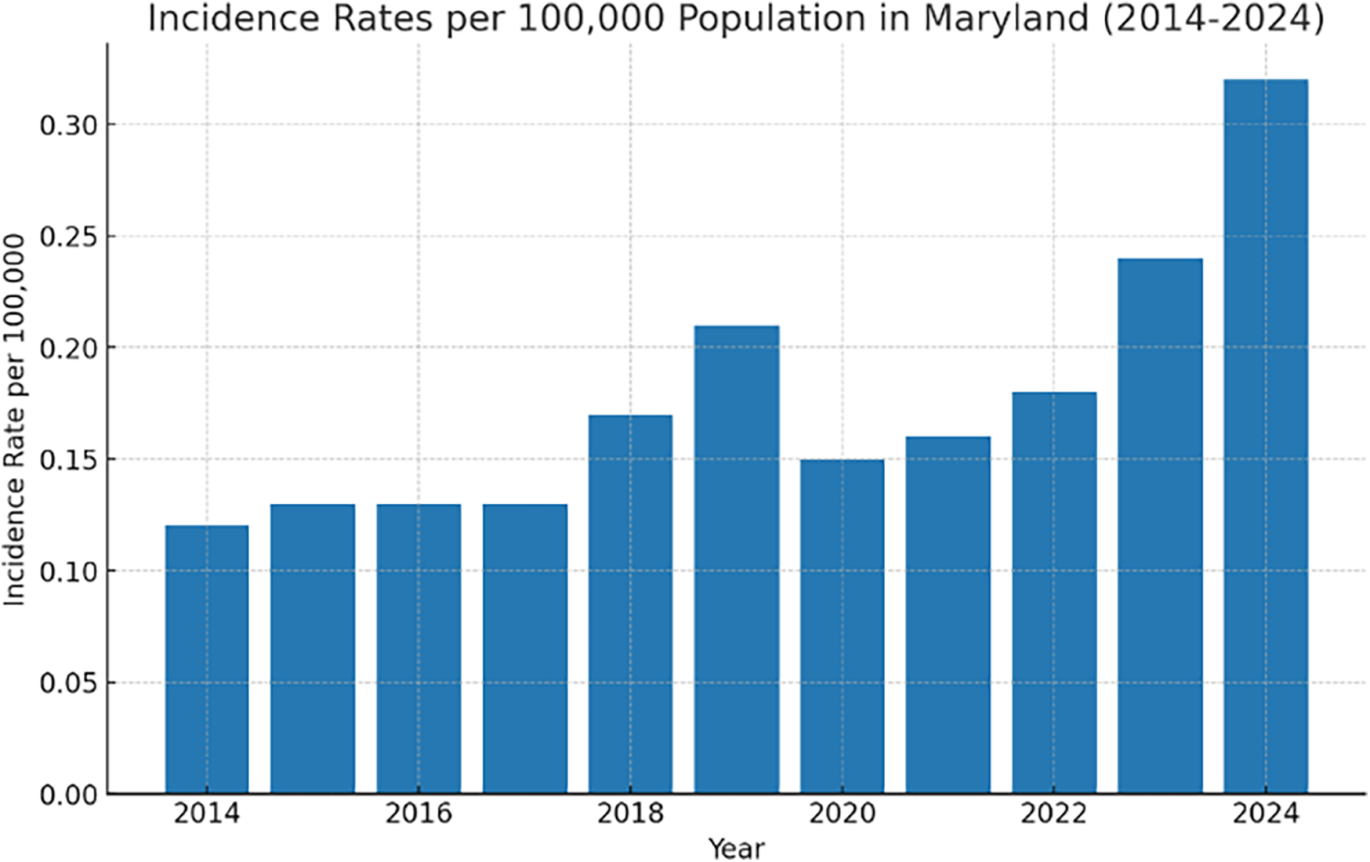
Dengue fever incidence rates per 100,000 population in Maryland sliced from 2014 to 2024.

Using the Maryland population data for each year, the incidence rates were calculated as follows (Figure 1):

• 2014: 0.12 per 100,000
• 2015: 0.13 per 100,000
• 2016: 0.13 per 100,000
• 2017: 0.13 per 100,000
• 2018: 0.17 per 100,000
• 2019: 0.21 per 100,000
• 2020: 0.15 per 100,000
• 2021: 0.16 per 100,000
• 2022: 0.18 per 100,000
• 2023: 0.24 per 100,000
• 6-month incidence of dengue in 2024, using the 2023 population data, is approximately 0.32 cases per 100,000 population

### Environmental data: temperature and humidity trends

Temperature and Humidity in July in Salisbury, Maryland, from 2014 to 2024 (16).

Salisbury is one of the major population centers in the eastern shore of Maryland.

Peak July Temperature Levels:

• 2014: 85°F
• 2015: 87°F
• 2016: 86°F
• 2017: 88°F
• 2018: 89°F
• 2019: 90°F
• 2020: 88°F
• 2021: 91°F
• 2022: 89°F
• 2023: 88°F
• 2024: 90°F

Peak July Humidity Levels (17):

• 2014: 83%
• 2015: 84%
• 2016: 84%
• 2017: 85%
• 2018: 86%
• 2019: 85%
• 2020: 84%
• 2021: 86%
• 2022: 85%
• 2023: 86%
• 2024: 86%

## Correlation analysis results

### Temperature

The relationship between peak July temperature and dengue fever incidence in Maryland was analyzed using a linear regression model, and multiple regression model. The model revealed a moderate positive correlation between peak July temperature and the incidence of dengue fever.

### Linear regression

#### Excluding pandemic years (2020 and 2021)

**Figure 2:**
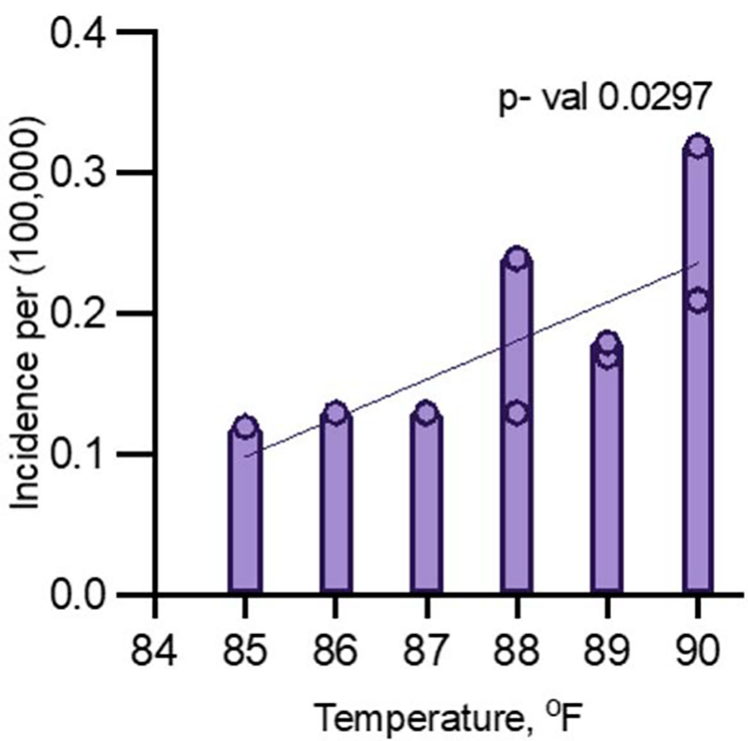
Multiple logistic regression analysis for dengue incidence and peak July temperature in Maryland from 2014 to 2024 (excluding 2020 and 2021).

The regression analysis, excluding 2020 and 2021, yielded an R-squared value of 0.5143, indicating that approximately 51.43% of the variance in dengue fever incidence could be explained by changes in peak July temperature (Figure 2). The F-statistic of 7.413 with a *p*-value of 0.0297 suggests that this relationship is statistically significant at the 5% level, indicating that peak July temperature is a significant predictor of dengue fever incidence in Maryland. However, the adjusted R-squared of 0.4449 indicates that after accounting for model complexity, the explained variance drops to 44.49%, highlighting that while temperature is an important factor, other environmental or socio-demographic variables likely contribute to the incidence of dengue fever (Figure 2).

#### Including pandemic years (2020 and 2021)

**Figure 3:**
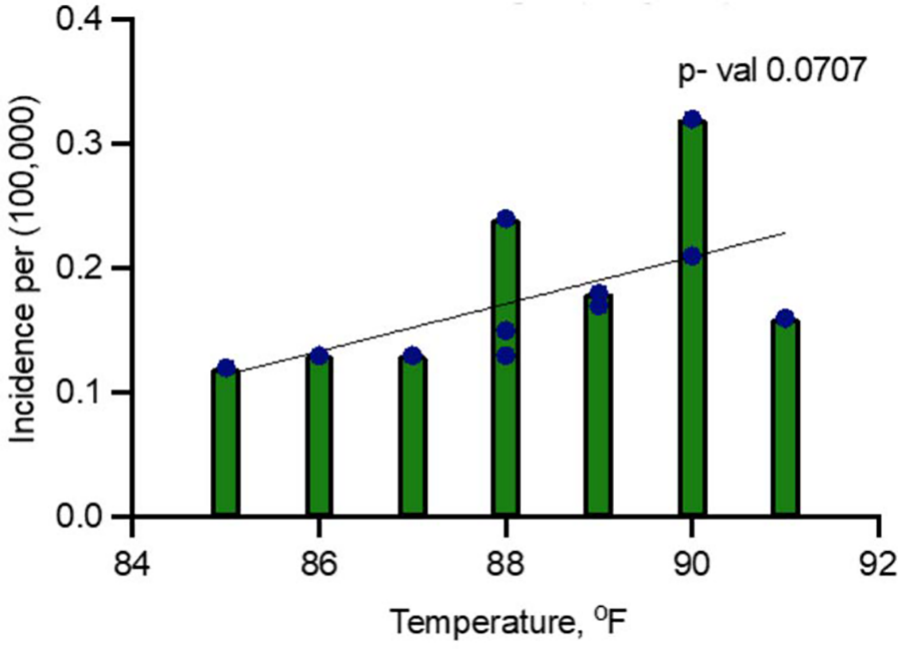
Linear regression analysis for dengue incidence and peak July temperature in Maryland from 2014 to 2024.

A second model, including all years (2014–2024), showed a positive relationship between temperature and dengue incidence, but it was not statistically significant. In this model, the R-squared value was 0.3181, indicating that only 31.81% of the variance in dengue fever incidence could be explained by peak July temperature (Figure 3). The F-statistic of 4.199 with a *p*-value of 0.0707 suggests that the relationship was not significant at the 5% level. Additionally, the adjusted R-squared was 0.2424, indicating that the model fit was weaker when the pandemic years were included (Figure 3).

### Multiple regression

A multiple regression analysis was conducted to determine the influence of peak July temperature and year on the incidence of dengue fever (per 100,000 population) in Maryland. The model aimed to assess how both predictors contribute to the variation in dengue fever incidence over time.

#### Including pandemic years (2020 and 2021)

Year was a statistically significant predictor of dengue incidence (*p* = 0.0124), indicating that the incidence of dengue fever has been increasing over time (Table 1). Temperature had a *p*-value of 0.0655, suggesting a potential trend, but it was not statistically significant at the 5% level. This indicates that while temperature is related to dengue incidence, other factors may also play a role. The F-statistic of 8.712 and the *p*-value of 0.0064 indicate that the overall model is statistically significant, meaning both predictors (temperature and year) together explain a substantial amount of variation in dengue incidence (Table 1).

**Table 1:** Correlation- multiple logistic regression analysis.

Correlation *p*-values	Year	Temperature	Incidence
Year (excluding 2020–2021)		0.0093	0.001
Temperature	0.0093		0.0297
Dengue incidence (per 100,000)	0.001	0.0297	

#### Excluding 2020 and 2021

The coefficient for year increased slightly to 0.0264, with a *p*-value of 0.0032, indicating that the effect of year remains statistically significant. The model fit improves, explaining 74.58% of the variation in dengue incidence, and temperature becomes a significant predictor. The coefficient for temperature increased to 0.0158, and the *p*-value of 0.0297 indicates that temperature becomes a statistically significant predictor when the pandemic years are excluded (Table 2).

**Table 2:** Correlation- multiple logistic regression analysis.

Correlation *p*-values	Year	Temperature	Incidence
Year (including 2020 and 2021)		0.012	0.0023
Temperature	0.012		0.0707
Dengue incidence (per 100,000)	0.0023	0.0707	

### Humidity

#### Linear regression

The linear regression analysis revealed a significant association between humidity and dengue incidence in Maryland from 2014 to 2024.

##### Including pandemic years (2020 and 2021)

**Figure 4:**
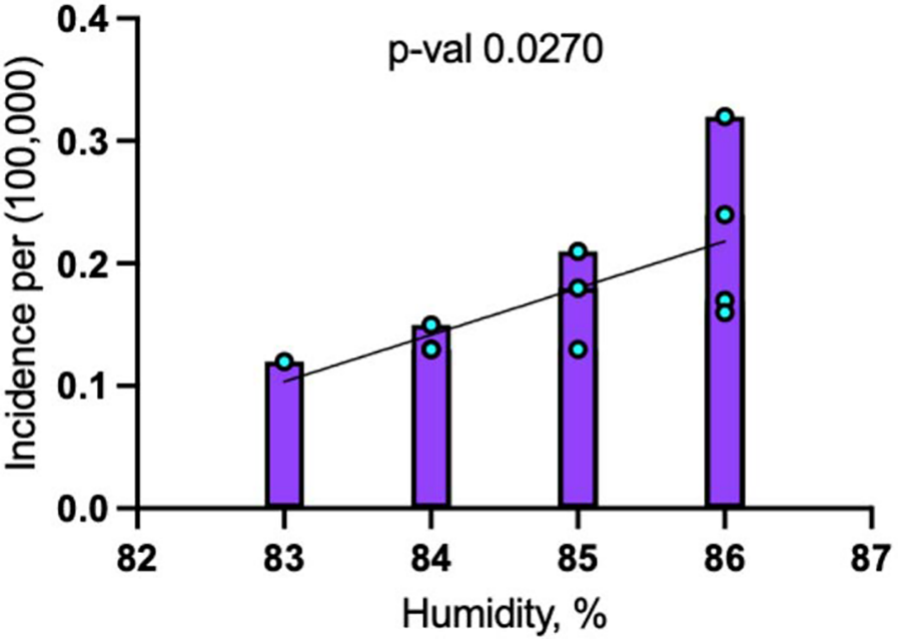
Linear regression analysis for dengue incidence and peak July humidity in Maryland from 2014 to 2024.

When including 2020 and 2021, the model showed that for every 1% increase in humidity, dengue incidence increased by 0.038 cases per 100,000 people (*p* = 0.0270). This model explained 43.6% of the variance in dengue incidence (R-squared = 0.4359), indicating a moderately strong relationship between humidity and disease occurrence (Figure 4).

##### Excluding pandemic years (2020 and 2021)

**Figure 5:**
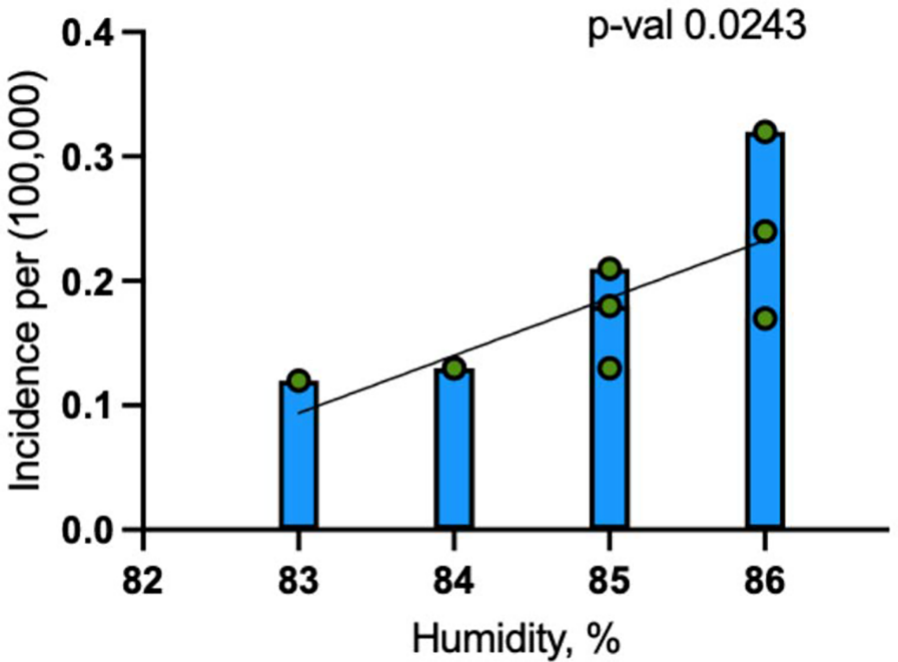
Linear regression analysis for dengue incidence and peak July humidity in Maryland from 2014 to 2024 (excluding 2020 and 2021).

When the pandemic years of 2020 and 2021 were excluded, the association between humidity and dengue incidence became stronger, with the coefficient increasing to 0.046 cases per 100,000 people for each 1% rise in humidity (*p* = 0.0243) (Figure 5). The model excluding these years explained 53.9% of the variance (R-squared = 0.5388), reflecting an improved fit.

## Discussion

As the climate in the United States changes, there is likely to be a continued expansion of dengue fever in the US. With global warming increasing the overall temperature in Maryland and the US, weather conditions will increase suitable for transmitting dengue fever (18). An adult Aedes mosquito's survival rate dramatically increases at temperatures above 20 degrees Celsius. The Aedes mosquitos are in the Southern US, as temperatures have increased, there is a concern for an increase in areas becoming endemic to Aedes mosquitoes with dengue (19, 20). This temperature change could potentially explain the increase in the number of cases of dengue in the US each year. As national temperatures increase, there is also an increase in the duration of time in which adult Aedes mosquitoes can live (21). With adult Aedes mosquitoes living longer in areas they had not before, there is an increase in potential vectors for the dengue virus, explaining an increase in dengue virus infections in the US. Between 1995 and 2016, 177 U.S. counties reported the presence of both Ae. Aegypti and Ae. Albopticus. Most of these counties were located in Maryland, southern California, Arizona, Texas, and Florida (22). Additionally, according to the CDC, autochthonous cases of dengue fever have occurred in Florida, Texas, Hawaii, Arizona, and California. Florida, Texas, and California, along with some states in the northeast (Virginia, Pennsylvania, New York) have seen an overall increase in dengue fever cases from 2014 to 2024 (23).

The CDC does not mention Maryland as one of the states in which autochthonous cases have occurred; however, this could be due to the possibility that surveillance measures in Maryland are not as extensive as in the states where autochthonous cases have occurred. This could potentially result in an underreporting of cases in Maryland.

It is possible that American travelers to regions where dengue is endemic can acquire the virus and upon returning to the US, serve as reservoirs that spread infection to Aedes mosquitoes (24). This can help explain the rising cases of dengue each year in the US.

The results of this study suggest a significant trend in dengue fever incidence in Maryland over the past decade. The prevalence of dengue fever increased over the study period, with annual cases as follows: 7 in 2014, with an incidence rate of 0.12 per 100,000, to a rise to 13 in 2019, with an incidence rate of 0.21 per 100,000. Despite the COVID-19 lockdown, which was expected to result in fewer cases due to restricted travel, the reduction in incidence was not as pronounced as anticipated. Following the lockdown, the number of cases increased, with 15 in 2023 and an incidence rate of 0.24 per 100,000, and it went up to 20 patients in the first 6 months of 2024 with an incidence rate of 0.32 cases per 100,000 population.

The linear regression analyses exploring the relationship between temperature and dengue incidence in Maryland revealed notable findings, particularly when comparing data that included and excluded the pandemic years 2020 and 2021. In the full dataset, temperature was positively correlated with dengue incidence, but the relationship was not statistically significant (*p* = 0.0707), suggesting that external factors during the pandemic, such as reduced travel, altered healthcare access, and changes in mosquito control, may have disrupted typical transmission patterns. However, when the pandemic years were excluded, the correlation became statistically significant (*p* = 0.0297), with temperature explaining 51% of the variability in dengue incidence. This indicates that temperature plays a significant role in dengue transmission under normal conditions, though external factors can obscure its influence.

The multiple logistic regression analysis indicates that both year and temperature are significantly associated with the incidence of dengue fever in Maryland when the years 2020 and 2021 are excluded. This suggests that the relationship is likely driven by the interplay between rising temperatures and normal dengue transmission dynamics. However, when these two pandemic years are included, the correlation between temperature and dengue incidence weakens (*p* = 0.0707). This implies that the pandemic may have affected the strength of this relationship, possibly due to changes in travel patterns, healthcare access, or vector control efforts during that period, which may have disrupted the usual transmission patterns.

These findings highlight the complexity of studying dengue transmission, as disruptions like the COVID-19 pandemic can mask environmental factors. Humidity, however, appears to have an even stronger influence on dengue transmission, particularly in regions like Maryland's Eastern Shore. The region's humid subtropical climate, influenced by the Chesapeake Bay and the Atlantic Ocean (25), creates favorable conditions for mosquito survival and dengue transmission, potentially explaining the disproportionately higher dengue cases with 24% of the dengue cases resided on the Eastern Shore of Maryland, despite this region representing only 7.4% of the state's population.

The warmer temperatures and the rise in temperature over time contributed to the expansion of mosquito habitats, particularly in temperate regions like Maryland, where previously inhospitable climates are becoming more conducive to mosquito proliferation (26).

First found in 1987, Aedes albopictus eggs are present year-round in Maryland. Larvae are typically present from April through October, while adult mosquitoes are found from May through October. The period of peak population is from June through September (27). The optimal temperature range for mosquito growth is 77–86°F (25–30°C), with humidity having a stronger correlation to transmission (28). At less than 60% humidity, the mosquitos have a short lifespan and do not become vectors (29). The mosquitoes survive longer at 85% humidity, with adequate time to transfer the virus from the stomach to the salivary glands of the female mosquitoes. Interestingly, there was no marked decline in the incidence of dengue cases in Maryland when travel restrictions were in place during the COVID-19 pandemic from 2020 to 2021. Although there were fewer international travelers (20), which should have limited the introduction of dengue into the state, the incidence remained relatively stable. This indicates that local mosquito populations may have been sufficient to maintain transmission or other factors such as domestic travel or undetected local cases contributed to the persistence of dengue.

Overall, the study emphasizes the need to address local environmental factors, particularly humidity, when managing and preventing dengue transmission in Maryland.

## Limitations

A key limitation of our study is the low overall number of dengue cases, which limits our ability to draw broad conclusions about long-term trends. Additionally, we were unable to differentiate between locally acquired and imported cases due to the absence of travel history data in the dataset. We also could not distinguish between confirmed and suspected cases, as we relied solely on ICD-10 codes for dengue diagnoses. Furthermore, we were unable to analyze dengue cases on a monthly or weekly basis, restricting our ability to explore potential seasonal variations. Future studies should focus on differentiating these cases and conducting in-depth analysis of local transmission patterns, particularly in regions like the Eastern Shore, where the prevalence is disproportionately high.

## Conclusion

While dengue fever cases have risen in Maryland, we cannot definitively conclude that this increase is due to endemic transmission within the state or climate change alone. The association between rising temperatures and dengue incidence, particularly when excluding the pandemic years, suggests that imported cases may play a significant role in local outbreaks.

Travel during peak seasons, combined with favorable humidity and temperature conditions, could facilitate autochthonous transmission, as seen in other regions like Australia and Europe. To address this issue, health authorities need to establish better surveillance of each dengue case, including thorough case investigations to trace travel histories and accurate diagnostics to confirm dengue infections.

Enhanced vector control and public health measures will be crucial in mitigating the future spread of dengue fever in Maryland. Our findings particularly underscore the necessity for heightened vector control and public health initiatives in humid regions like the Eastern Shore, where environmental factors strongly influence dengue transmission.

## Data Availability

The original contributions presented in the study are included in the article/Supplementary Material, further inquiries can be directed to the corresponding authors.

## References

[1] Murugesan A Manoharan M. Dengue virus. Emerg Reemerg Viral Pathogens. (2020) 1:281–359. 10.1016/B978-0-12-819400-3.00016-8

[2] Brathwaite Dick O San Martín JL Montoya RH del Diego J Zambrano B Dayan GH. The history of dengue outbreaks in the americas. Am J Trop Med Hyg. (2012) 87(4):584–93. 10.4269/ajtmh.2012.11-0770 23042846 PMC3516305

[3] Schaefer TJ Panda PK Wolford RW. Dengue fever. In: StatPearls. Treasure Island, FL: StatPearls Publishing (2024). Available online at: http://www.ncbi.nlm.nih.gov/books/NBK430732/ (cited August 06, 2024). 28613483

[4] Dengue and severe dengue. Available online at: https://www.who.int/news-room/fact-sheets/detail/dengue-and-severe-dengue (cited August 06 2024).

[5] Adalja AA Sell TK Bouri N Franco C. Lessons learned during dengue outbreaks in the United States, 2001–2011. Emerg Infect Dis. (2012) 18(4):608–14. 10.3201/eid1804.110968 22469195 PMC3309700

[6] Moore CG Mitchell CJ. Aedes albopictus in the United States: ten-year presence and public health implications. Emerg Infect Dis. (1997) 3(3):329–34. 10.3201/eid0303.970309 9284377 PMC2627635

[7] Faiman R Goodwin A Cave-Stevens J Schultz A Brey J Ford T. Aedes aegypti in Maryland: The need for elevated vector surveillance at the face of a dynamic climate. *bioRxiv: 2023.10.02.560479* (2023). Available from: http://biorxiv.org/content/early/2023/10/03/2023.10.02.560479.abstract

[8] Rothman SE Jones JA LaDeau SL Leisnham PT. Higher west Nile virus infection in Aedes albopictus (Diptera: culicidae) and culex (Diptera: culicidae) mosquitoes from lower income neighborhoods in urban Baltimore, MD. J Med Entomol. (2021) 58(3):1424–8. 10.1093/jme/tjaa262 33257956

[9] Vijay J Anuradha N Anbalagan VP. Clinical presentation and platelet profile of dengue fever: a retrospective study. Cureus. (2022) 14(8):e28626. Available online at: https://www.ncbi.nlm.nih.gov/pmc/articles/PMC9524240/36196330 10.7759/cureus.28626 PMC9524240

[10] CDC. Dengue. Clinical Testing Guidance for Dengue (2024). Available online at: https://www.cdc.gov/dengue/hcp/diagnosis-testing/index.html (cited August 06, 2024).

[11] Dengue Transmission | Learn Science at Scitable. Available online at: https://www.nature.com/scitable/topicpage/dengue-transmission-22399758/ (cited August 06, 2024).

[12] Chishtie J Sapiro N Wiebe N Rabatach L Lorenzetti D Leung AA Use of epic electronic health record system for health care research: scoping review. J Med Internet Res. (2023) 25:e51003. 10.2196/51003 38100185 PMC10757236

[13] *Maryland Population 1900-*2023. macrotrends.net (2024). Available online at: https://www.macrotrends.net/global-metrics/states/maryland/population (cited October 10, 2024).

[14] Maryland Population 2024 (Demographics, Maps, Graphs). worldpopulationreview.com (2024). Available online at: https://worldpopulationreview.com/states/maryland (cited October 10, 2024).

[15] U.S. Census Bureau. *Explore Census Data*. census.gov (2024). Available online at: https://data.census.gov/profile/Maryland?g=0400000US24 (cited October 10, 2024).

[16] Cedar Lake Ventures, Inc. Average Weather in Salisbury, Maryland, United States - Year Round. WeatherSpark. Available online at: https://weatherspark.com/y/22683/Average-Weather-in-Salisbury-Maryland-United-States-Year-Round#Sections-Temperature (cited October 10, 2024).

[17] WeatherSpark. Average Weather in Salisbury, Maryland, United States - Year Round. Cedar Lake Ventures, Inc. Available online at: https://weatherspark.com/y/22683/Average-Weather-in-Salisbury-Maryland-United-States-Year-Round#Sections-Humidity (cited October 10, 2024).

[18] Laporta GZ Potter AM Oliveira JFA Bourke BP Pecor DB Linton YM. Global distribution of Aedes aegypti and Aedes albopictus in a climate change scenario of regional rivalry. Insects. (2023) 14(1):49. 10.3390/insects14010049 36661976 PMC9860750

[19] Maryland - Coronavirus State Actions. National Governors Association (2020). Available online at: https://www.nga.org/coronavirus-state-actions/maryland/ (cited August 06, 2024).

[20] Shemer L Shayanfar E Avner J Miquel R Mishra S Radovic M. COVID-19 impacts on mobility and travel demand. Case Stud Transp Policy. (2022) 10(4):2519–29. 10.1016/j.cstp.2022.11.011 36407477 PMC9661421

[21] Mulderij-Jansen V Pundir P Grillet ME Lakiang T Gerstenbluth I Duits A Effectiveness of aedes-borne infectious disease control in Latin America and the Caribbean region: a scoping review. PLoS One. (2022) 17(11):e0277038. 10.1371/journal.pone.0277038 36322603 PMC9629598

[22] Hahn MB Eisen L McAllister J Savage HM Mutebi JP Eisen RJ. Updated reported distribution of aedes (stegomyia) aegypti and aedes (stegomyia) albopictus (Diptera: culicidae) in the United States, 1995–2016. J Med Entomol. (2017) 54(5):1420. 10.1093/jme/tjx088 28874014 PMC5968631

[23] Data and Statistics on Dengue in the United States | Dengue | CDC. Available online at: https://www.cdc.gov/dengue/data-research/facts-stats/index.html (cited October 18, 2024).

[24] Current and Projected Distributions of Aedes aegypti and Ae. albopictus in Canada and the U.S. | Environmental Health Perspectives | Vol. 128, No. 5. Available online at: https://ehp.niehs.nih.gov/doi/10.1289/EHP5899 (cited October 18, 2024). 10.1289/EHP5899 PMC7263460 32441995

[25] Bready JH DiLisio JE. “Maryland”. Encyclopedia Britannica (2024). Available online at: https://www.britannica.com/place/Maryland-state (Accessed October 10, 2024).

[26] Wells CR Gokcebel S Pandey A Galvani AP Townsend JP. Testing for COVID-19 is much more effective when performed immediately prior to social mixing. Int J Public Health. (2022) 67:1604659. 10.3389/ijph.2022.1604659 35967267 PMC9363582

[27] *Mosquito Control Program Description*. Maryland.gov Enterprise Agency Template (2024). Available online at: https://mda.maryland.gov/plants-pests/Pages/default.aspx (cited October 10, 2024).

[28] Monintja TC Arsin AA Amiruddin R Syafar M. Analysis of temperature and humidity on dengue hemorrhagic fever in manado municipality. Gaceta Sanitaria. (2021) 35:S330–3. 10.1016/j.gaceta.2021.07.020 34929845

[29] Rocklöv J Tozan Y. Climate change and the rising infectiousness of dengue. Emerg Top Life Sci. (2019) 3:133–42. 10.1042/ETLS20180123 33523146 PMC7288996

